# Histological and Strand-Specific Transcriptomic Analyses Reveal Developmental Dynamics and Pigmentation-Associated Candidate Genes in Black and White Skin Regions of Bama Miniature Pigs

**DOI:** 10.3390/ani16162565

**Published:** 2026-08-17

**Authors:** Lingli Feng, Qin Xia, Wenwen Xu, Zhongrong Deng, Alida Abla, Jiafu Li, Jinning Liang, Yanping Zhang, Xiaoping Guo, Jing Liang

**Affiliations:** 1 Liangzhu Laboratory, Zhejiang University School of Medicine, Zhejiang University, Hangzhou 311100, China; 15678852652@163.com; 2 Guangxi Key Laboratory of Animal Breeding and Disease Control, College of Animal Science and Technology, Guangxi University, Nanning 530004, China; qinxia1994@hotmail.com (Q.X.); m15213036981@163.com (Z.D.); alida_abla@163.com (A.A.); zyp122659@163.com (Y.Z.); 3 Institute of Animal Husbandry, Guangxi Vocational University of Agriculture, Nanning 530007, China; xww920730@outlook.com; 4 Laboratory Animal Center, Guangxi Medical University, Nanning 530021, China; 15676788941@163.com (J.L.); liangjinning2026@163.com (J.L.)

**Keywords:** Bama miniature pig, black skin, white skin, RNA-seq, pigmentation

## Abstract

Guangxi Bama miniature pigs are a highly inbred population developed from Bama Xiang pigs and exhibit a stable “two-end black” skin phenotype, making them a useful model for studying regional skin pigmentation. However, the developmental timing and molecular basis of this color difference remain unclear. In this study, black and white skin samples were collected from the posterior head/neck region at six developmental stages: embryonic days 65, 90, and 105, birth, and postnatal days 30 and 180. Histological examination and strand-specific RNA sequencing showed that developmental stage was the major factor shaping skin gene expression, whereas black–white skin differences varied across stages. Transcriptomic differences were already detectable at embryonic day 65, although visible regional differences were not obvious. Pigmentation-associated genes, including *DCT*, *SLC24A5*, *TRPM1*, and *TYRP1*, showed black-skin-biased expression during embryonic development, especially at embryonic day 105. Histological analysis also revealed progressive skin maturation and qualitative differences in darkly stained structures between black and white skin. These findings suggest that molecular differences between black and white skin emerge during embryonic development and persist after birth, providing resources and candidate genes for studying regional pigmentation in Bama miniature pigs.

## Simple Summary

Guangxi Bama miniature pigs have a stable genetic background and a typical “two-end black” coat pattern, with black skin at both ends of the body and white skin in the middle. This makes them a useful animal model for studying how regional coat color differences form within the same breed. However, it remains unclear when these black and white skin differences appear during development and which biological changes are involved. In this study, black and white skin samples were collected from the posterior head/neck region of Guangxi Bama miniature pigs at six developmental stages, from embryonic day 65 to 180 days after birth. We examined skin structure and analyzed changes in genes related to skin development and pigmentation. The results showed that developmental stage was the main factor shaping the skin transcriptome and that differences between black and white skin varied in a stage-dependent manner. Pigmentation-associated gene expression differences became more prominent during late embryonic development, especially at embryonic day 105. These findings suggest that molecular differences between black and white skin are detectable before birth and persist after birth. This study helps explain regional coat color formation in miniature pigs and provides useful information for animal breeding, genetics, and pigmentation research.

## 1. Introduction

Skin and coat pigmentation are important phenotypic traits in livestock and are widely used for breed identification, genetic resource characterization, environmental adaptation studies, and selective breeding [1]. In domestic pigs, coat-color patterns have been strongly shaped by long-term artificial selection and are widely used for breed characterization and conservation. Beyond their value as breed-specific external phenotypes, pigmentation traits may also be associated with environmental adaptation in some livestock species. For example, coat color has been associated with differences in cold tolerance in dairy cattle, suggesting that pigmentation may influence physiological adaptation to environmental conditions [2]. Furthermore, pigmentation is not always a fixed phenotype. Juvenile wild boars display characteristic striped camouflage coats that gradually disappear during postnatal development, indicating that coat-color formation is a dynamic developmental process rather than a static adult trait [3]. Nevertheless, most livestock pigmentation studies have focused on genetic loci [4], selection signatures, or transcriptomic differences between adult pigmentation phenotypes [5,6], whereas relatively few studies have investigated the developmental timing and molecular programs underlying the establishment of regional skin pigmentation.

In pigs, as in other mammals, skin and coat pigmentation are primarily determined by the type, amount, and distribution of melanin synthesized by melanocytes. Melanocytes originate from neural crest-derived precursors and are mainly located in the basal layer of the epidermis and hair follicles, where they produce melanin within specialized organelles known as melanosomes [7,8]. Pigmentation depends on a coordinated series of biological processes, including melanocyte differentiation and migration, melanosome biogenesis and maturation, melanin biosynthesis, and melanosome transfer to surrounding keratinocytes [9]. At the molecular level, *MITF* acts as a central transcriptional regulator of melanocyte development and melanogenesis [10], integrating upstream signals mediated by factors such as *SOX10* and *PAX3*, as well as upstream signaling pathways mediated by *MC1R* [11] and *KIT* [12,13], and regulating downstream melanogenic enzymes, including *TYR*, *TYRP1*, and *DCT* [14,15]. In parallel, melanosome-associated and trafficking genes, such as *PMEL* [16], *MLANA*, *RAB27A*, and *MLPH* [17], contribute to melanosome maturation, transport, and pigment distribution [18,19]. In pigs, classical genetic studies have identified *MC1R* [20] and *KIT* as key genes influencing coat-color variation and color-pattern formation across breeds [21,22]. Together, these findings indicate that pig skin and coat-color phenotypes are shaped by coordinated regulation of melanocyte development, melanin synthesis, and melanosome transfer.

The Guangxi Bama miniature pig is a Chinese inbred miniature pig population with a stable “two-end black” phenotype [23], in which the skin at both ends of the body is black whereas the middle region is white. This naturally occurring black–white contrast provides a valuable system for investigating regional pigmentation-associated skin differences within the same breed. Previous transcriptomic analysis of adult Bama pig skin showed that white skin lacks melanocytes whereas black skin contains melanocytes, and identified differentially expressed mRNAs and lncRNAs associated with pigmentation, keratinization, melanocyte–keratinocyte interaction, and energy metabolism [24]. Consistently, studies of Chinese indigenous pigs with regional black–white coat-color patterns, including black-back/white-belly Wuzhishan pigs, have shown that both genetic variation and coding/non-coding transcriptional regulatory networks contribute to regional pigmentation differences [25]. Large-scale genomic analyses of Chinese indigenous pigs further suggest that coat-color differentiation in these breeds may involve loci beyond the classical *MC1R* and *KIT* genes, with *EDNRB* emerging as an important candidate in several Chinese pig populations [26,27]. Together, previous studies have mainly focused on adult skin, genetic association analyses, or comparisons of already established pigmentation phenotypes. However, when differences between black and white skin appear during development in Bama miniature pigs and which transcriptional changes are associated with them remain unclear.

To address this gap, we collected paired black and white skin samples from Bama miniature pigs at six developmental stages, including E65, E90, E105, D0, D30, and D180, and integrated histological examination, strand-specific RNA sequencing, time-course expression profiling, co-expression network analysis, and qRT-PCR analysis to systematically characterize the developmental changes and transcriptional features associated with the establishment of black–white skin differences. At each developmental stage, paired black and white skin samples were collected from the same female animal to minimize the effects of inter-individual genetic background and sex-related variation. This study aimed to describe the developmental dynamics of black and white skin differences in Bama miniature pigs, identify candidate genes associated with black–white transcriptional differences and skin pigmentation, and provide a developmental transcriptomic resource for further understanding the mechanisms of regional skin pigmentation in Bama miniature pigs.

## 2. Materials and Methods

### 2.1. Ethics Statement

All animal procedures were performed in accordance with the guidelines for the care and use of experimental animals and were approved by the Institutional Animal Care and Use Committee of Guangxi University (GXU2018-061).

### 2.2. Animals, Developmental Stages, and Sample Collection

**Figure 1:**
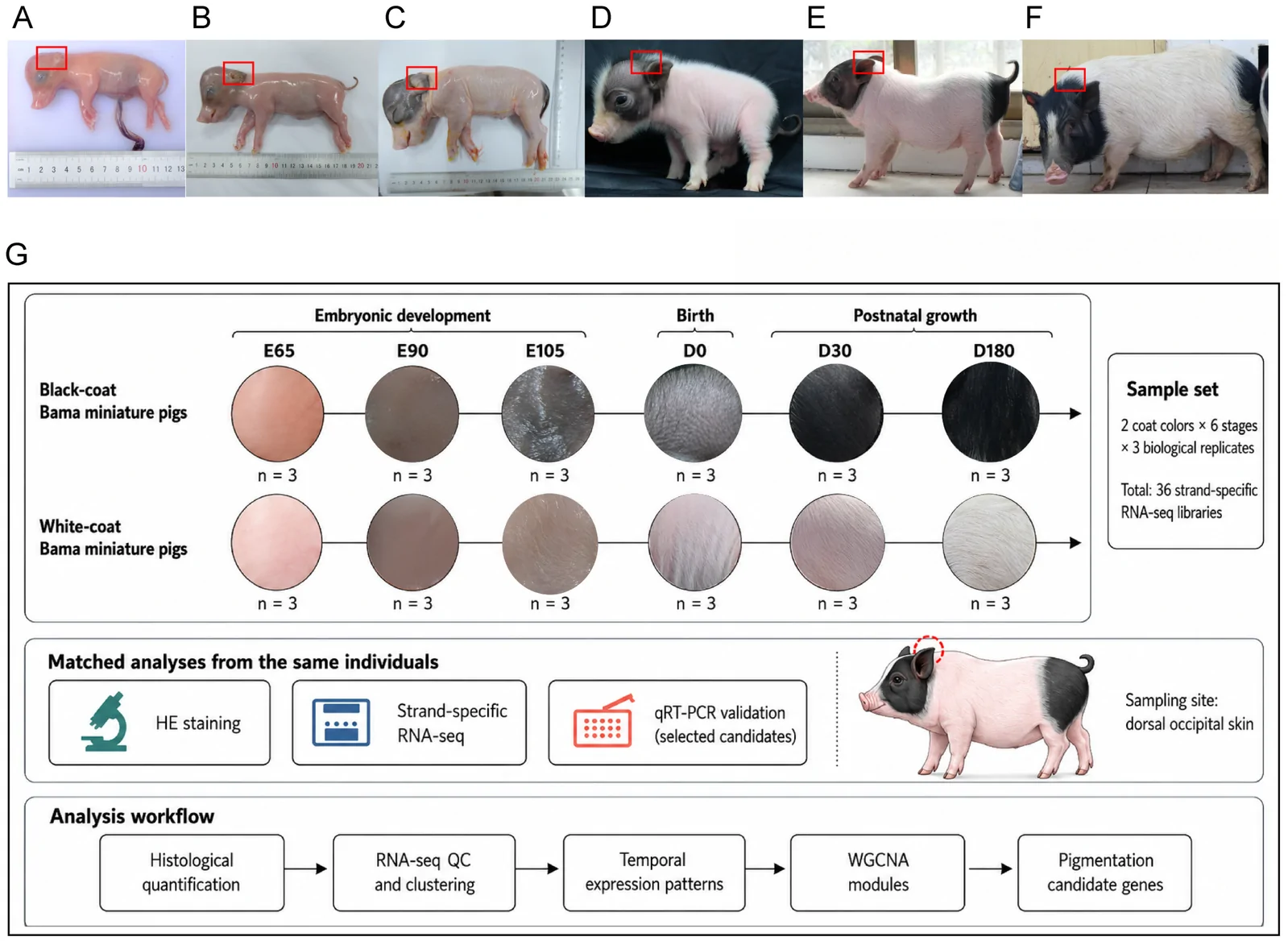
Gross morphology, skin sampling sites, and overall experimental design of Guangxi Bama miniature pigs at different developmental stages. (**A**–**F**) Representative images of Guangxi Bama miniature pigs from E65 to D180, with red rectangles marking the posterior head/neck skin sampling regions. (**G**) Schematic diagram of the study design, including the collection of paired black and white skin samples from the posterior head/neck region of Guangxi Bama miniature pigs at six developmental stages from E65 to D180, followed by H&E staining, strand-specific RNA-seq, and qRT-PCR analysis.

Guangxi Bama miniature pigs were obtained from the Bama Miniature Pig Breeding Center of Guangxi University. A total of 18 female animals were included, with three animals serving as independent biological replicates at each of the six developmental stages: embryonic day 65 (E65), embryonic day 90 (E90), embryonic day 105 (E105), postnatal day 0 (D0), postnatal day 30 (D30), and postnatal day 180 (D180). Embryonic stages were defined as gestational days after natural mating, whereas postnatal stages were defined as days after normal birth. D0 samples were collected within 24 h after birth. Body weight information for Guangxi Bama miniature pigs at different developmental stages is provided in Supplementary Table S1. The developmental stages, gross morphology, skin sampling sites, and overall experimental design are summarized in Figure 1.

Paired black and white skin samples were collected from the posterior head/neck region of each animal, yielding three paired biological replicates per developmental stage and 36 skin samples in total. Samples were collected from clearly distinguishable black and white skin regions, avoiding transitional boundary areas whenever possible. The overall sampling design, which combined within-animal paired sampling from the same anatomical region with the use of female animals only, was designed to reduce variation associated with genetic background, sex, inter-individual variation, and anatomical site. Animals were euthanized by exsanguination following approved procedures, and skin tissues were collected immediately after death. Samples for RNA extraction were rapidly frozen in liquid nitrogen and stored at −80 °C. Samples for histological analysis were fixed in 10% neutral buffered formalin as described below.

### 2.3. RNA Extraction, Library Construction, and Sequencing

Total RNA was extracted from skin tissues using TRIzol reagent according to the manufacturer’s instructions (TaKaRa, Beijing, China). RNA purity and concentration were assessed using a Q5000 micro-volume UV–Vis spectrophotometer (Quawell Technology Inc., San Jose, CA, USA). RNA integrity was evaluated by agarose gel electrophoresis using agarose purchased from Beijing Solarbio Science & Technology Co., Ltd. (Beijing, China). Only high-quality RNA samples were used for library construction. Strand-specific RNA-seq libraries were constructed following standard protocols, including mRNA enrichment, fragmentation, cDNA synthesis, and PCR amplification. The libraries were sequenced on an Illumina NovaSeq 6000 platform (Illumina, Inc., San Diego, CA, USA) to generate 150-bp paired-end reads. Library preparation and sequencing were performed by Novogene Co., Ltd. (Beijing, China).

### 2.4. RNA-Seq Data Processing

For RNA-seq analysis, three biological replicates were included for each skin color at each developmental stage. Raw paired-end sequencing reads were processed to remove adapters and low-quality sequences to obtain clean reads. An entire read pair was discarded if either read contained adapter sequences, if ambiguous nucleotides (N) accounted for more than 10% of the read length, or if bases with a Phred quality score of ≤5 accounted for more than 50% of the read. Q20, Q30, GC content, and the sequencing error rate were calculated for the clean reads, which were used for all subsequent analyses. Clean reads were aligned to the Sus scrofa reference genome Sscrofa11.1 using HISAT2 v2.2.1. SAMtools v1.9 was used to process the alignment files and remove reads that were not successfully mapped to the reference genome. Transcript assembly and mRNA expression quantification were performed using StringTie v2.2.1 based on the corresponding genome annotation. Gene expression levels were calculated as fragments per kilobase of transcript per million mapped reads (FPKM), and gene-level count matrices were generated for differential expression analysis. For sample-level quality assessment, pairwise sample correlation, principal component analysis, and hierarchical clustering were performed using normalized expression matrices.

### 2.5. Differential Expression and Functional Enrichment Analysis

Differentially expressed genes (DEGs) were identified using DESeq2 (v1.44.0) based on raw read counts, with Benjamini–Hochberg-adjusted *p* value (FDR) < 0.05 and |log2FC| ≥ 1 as the significance criteria. For each developmental stage, paired black and white skin samples were compared to identify black–white DEGs within that stage. Animal identity was included as a blocking factor, and skin color was included as the variable of interest in the DESeq2 design (design = ~animal + color). Thus, the three paired black and white skin samples at each developmental stage were analyzed as paired biological replicates. To characterize developmental expression changes within each skin type, comparisons between adjacent developmental stages were performed separately for black and white skin. Because different animals were sampled at different developmental stages, these comparisons were treated as independent-group comparisons rather than repeated-measures comparisons. Functional annotation and enrichment analyses of DEGs were performed using DAVID (https://davidbioinformatics.nih.gov/) and the clusterProfiler R package (v4.12.6). Gene Ontology (GO) terms and Kyoto Encyclopedia of Genes and Genomes (KEGG) pathways were analyzed to characterize the biological functions and pathways associated with the DEGs. Enriched GO terms and KEGG pathways with a Benjamini–Hochberg-adjusted *p* value < 0.05 were considered statistically significant.

### 2.6. Time-Course Expression Profiling

To investigate developmental expression trajectories, time-course expression profiling was performed across the six developmental stages. Genes showing differential expression between black and white skin or marked temporal variation during development were selected for time-course expression profiling. Expression values were scaled by gene across stages, and temporal expression patterns were clustered using the ClusterGVis R package (v0.1.4). Gene clusters were defined according to their dynamic expression in black skin, and the corresponding expression patterns of the same gene sets were further examined in white skin to compare developmental trends between the two skin colors. Functional enrichment analysis was performed for each cluster to identify biological processes and pathways associated with pigmentation, melanogenesis, epidermal development, extracellular matrix remodeling, and skin maturation.

### 2.7. Weighted Gene Co-Expression Network Analysis

Weighted gene co-expression network analysis (WGCNA) [28] was performed using the WGCNA R package (v1.74.0) to identify co-expression modules associated with developmental stage and skin color. The expression matrix from all 36 RNA-seq samples was used as input. Before network construction, lowly expressed genes were removed by retaining genes with FPKM > 1 in at least six samples. Gene variability was then assessed using the median absolute deviation (MAD), and the top 8000 most variable genes were selected. In addition, all 26 predefined canonical pigmentation-related genes were retained in the WGCNA input after expression filtering to ensure coverage of known pigmentation pathways. A signed co-expression network was constructed using biweight midcorrelation (bicor), with maxPOutliers set to 0.1. The soft-thresholding power was evaluated by jointly considering the scale-free topology fit index and mean connectivity across the tested candidate powers. Although the scale-free topology fit index did not reach 0.8 within the tested range, a soft-thresholding power of 14 was selected because further increases in power resulted in progressively smaller improvements in the scale-free topology fit, whereas mean connectivity continued to decrease. Thus, power 14 was considered an appropriate balance between approximation to scale-free topology and preservation of network connectivity (Figure S2A). Network construction was performed using blockwiseModules with a signed topological overlap matrix, minModuleSize = 30, deepSplit = 2, pamRespectsDendro = FALSE, and mergeCutHeight = 0.25.

Module eigengenes were calculated to summarize the expression profile of each module. Module–trait associations were assessed using Pearson correlation between module eigengenes and sample traits, including developmental stage and skin color. Grey modules were excluded from biological interpretation. Hub genes within each module were ranked according to the absolute value of module membership, also referred to as eigengene-based connectivity (kME), which was calculated using bicor. Functional enrichment analysis of module genes was performed using the clusterProfiler R package (v4.12.6) for GO terms and KEGG pathways.

### 2.8. Gene Set Variation Analysis

Gene set variation analysis (GSVA) was performed using the GSVA R package (v1.52.3) to evaluate pathway-level activity across samples. The log2(FPKM + 1)-transformed expression matrix was used as input. For targeted analysis, three curated gene sets were included: pigmentation/melanogenesis-related genes, melanosome/organelle-related genes, and extracellular matrix/WNT remodeling-related genes. These gene sets were used to represent pigmentation-associated transcriptional activity, melanosome biology, and skin remodeling during development. GSVA scores were calculated using a Gaussian kernel. For each gene set, scores were summarized by developmental stage and skin color and visualized as mean ± SEM. Black–white differences in pathway activity across developmental stages were evaluated based on GSVA scores.

In addition, an unbiased pathway-level analysis was performed using human MSigDB Hallmark gene sets obtained through the msigdbr R package (v26.1.0). Human Hallmark genes were converted to their corresponding *Sus scrofa* orthologs before GSVA. The 50 Hallmark gene sets contained 4384 unique human genes, of which 4202 were mapped to 4202 unique pig orthologs. After intersection with the pig expression matrix, 3871 pig orthologs were retained. Gene sets with 10–300 detected genes were retained for analysis. All 50 Hallmark gene sets met this criterion, with 31–186 detected pig genes per gene set and a median of 155 genes. Stage-specific black–white differences in GSVA scores were calculated to identify pathways showing dynamic differences in pathway activity during skin development.

### 2.9. qRT-PCR Validation

Quantitative real-time PCR (qRT-PCR) was performed to experimentally assess differences in the expression of selected pigmentation-associated candidate genes between black and white skin. cDNA was synthesized from total RNA using the PrimeScript™ RT reagent Kit (Perfect Real Time; TaKaRa Bio, Beijing, China) according to the manufacturer’s instructions. qRT-PCR was performed using TB Green^®^ Premix Ex Taq™ II (TaKaRa Bio, Beijing, China) on a CFX96 Real-Time PCR System (Bio-Rad Laboratories, Inc., Hercules, CA, USA). Gene-specific primers were designed using Oligo 7.0 and synthesized by Sangon Biotech Co., Ltd. (Shanghai, China). Primer sequences are listed in Supplementary Table S2. *ACTB* (β-actin) was used as the internal reference gene, and relative gene expression levels were calculated using the 2^−ΔΔCt^ method. Each reaction was performed in triplicate, and for each biological sample, the mean Ct value of the three technical replicates for the target and reference genes were used to calculate ΔCt and relative expression. Three paired biological replicates were analyzed at each included developmental stage: E90, E105, D0, D30, and D180. E65 was not included in the qRT-PCR validation because no RNA, cDNA, or tissue material from this stage remained after completion of the RNA-seq and related analyses.

### 2.10. Histological Analysis

Black and white skin samples of approximately 0.5 × 0.5 × 0.2 cm were collected from the posterior head/neck region. Surface hair was removed, and samples were rinsed with sterile saline. Tissues were fixed in 10% neutral buffered formalin for 24 h, followed by routine paraffin embedding and sectioning at a thickness of 6 μm. Sections were stained with hematoxylin and eosin (H&E) and examined under a light microscope. For each sample, multiple non-overlapping fields were examined to assess skin morphology. H&E-stained sections were qualitatively examined for general skin morphology, including epidermal organization, dermal architecture, and hair follicle development. Representative images were selected to illustrate the histological characteristics of black and white skin across developmental stages.

### 2.11. Statistical Analysis

Microsoft Excel 2019 was used only for data organization and tabulation. Statistical analyses of the qRT-PCR data were performed using GraphPad Prism 8.0.2 (GraphPad Software, San Diego, CA, USA). qRT-PCR data are presented as the mean ± standard error of the mean (SEM). For each candidate gene, qRT-PCR data were analyzed using a two-way mixed-design ANOVA, with developmental stage as the between-subjects factor and skin color as the within-subjects repeated-measures factor. Three pigs were included as biological replicates at each developmental stage, and black and white skin measurements were paired within each pig. The model included the main effects of developmental stage and skin color, as well as their interaction. Bonferroni-adjusted pairwise comparisons were subsequently performed to compare paired black and white skin samples within each developmental stage. Statistical significance was defined as *p* < 0.05. For transcriptomic analyses involving multiple testing, *p* values were adjusted using the Benjamini–Hochberg method, and adjusted *p* values < 0.05 were considered statistically significant unless otherwise stated.

## 3. Results

### 3.1. Histological Features of Skin Development

**Figure 2:**
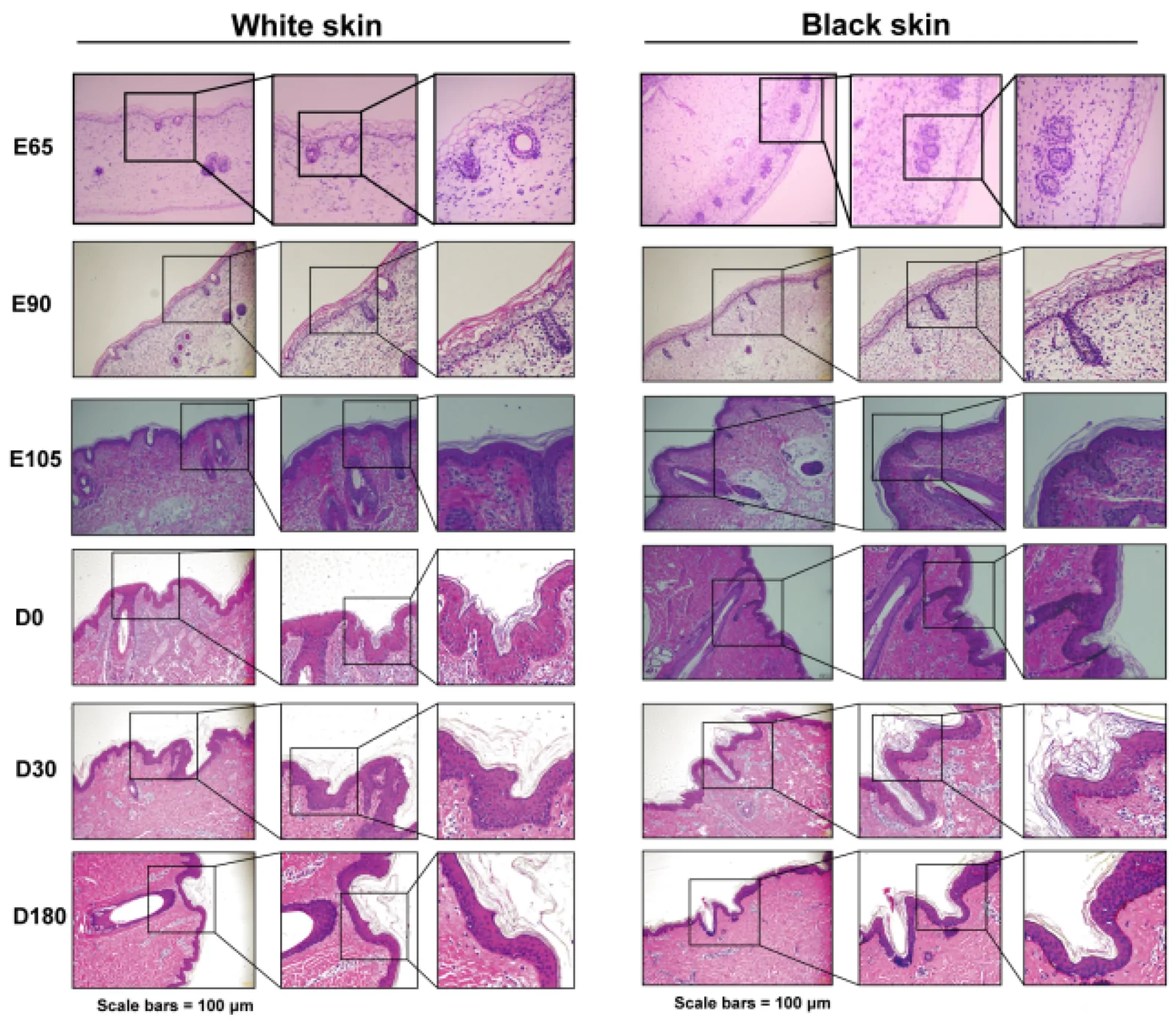
Histological characterization of paired black and white skin during development in Bama miniature pigs. Representative H&E-stained sections of black and white skin across developmental stages from E65 to D180. Boxed regions indicate areas shown at higher magnification in the adjacent images. Scale bars = 100 μm.

H&E staining showed that the skin structure of Guangxi Bama miniature pigs gradually matured during development, as reflected by increasingly distinct epidermal stratification, progressive organization of the dermis, and the formation and maturation of hair follicles (Figure 2). At embryonic day 65, the skin remained relatively immature, with a thin epidermis, loose dermal mesenchyme, and early hair follicle-like structures. The distribution of darkly stained structures differed between black and white skin regions, with these structures observed in the epidermis and hair follicle-associated areas of black skin. However, because H&E staining is not melanin-specific, the identity of these darkly stained structures could not be conclusively determined. At E90 and E105, epidermal stratification became more apparent and hair follicles continued to develop. During this period, darkly stained structures were observed in black skin regions around developing hair follicles and in the adjacent epidermis. At birth and during postnatal development, the epidermis, dermis, and hair follicles of both black and white skin continued to mature. Darkly stained structures continued to be observed in black skin, particularly in the epidermis, hair follicles, and perifollicular regions. Together, these observations showed progressive maturation of both black and white skin, accompanied by differences in the distribution of darkly stained structures between the two skin regions.

### 3.2. Transcriptomic Landscape of Black and White Skin Samples

**Figure 3:**
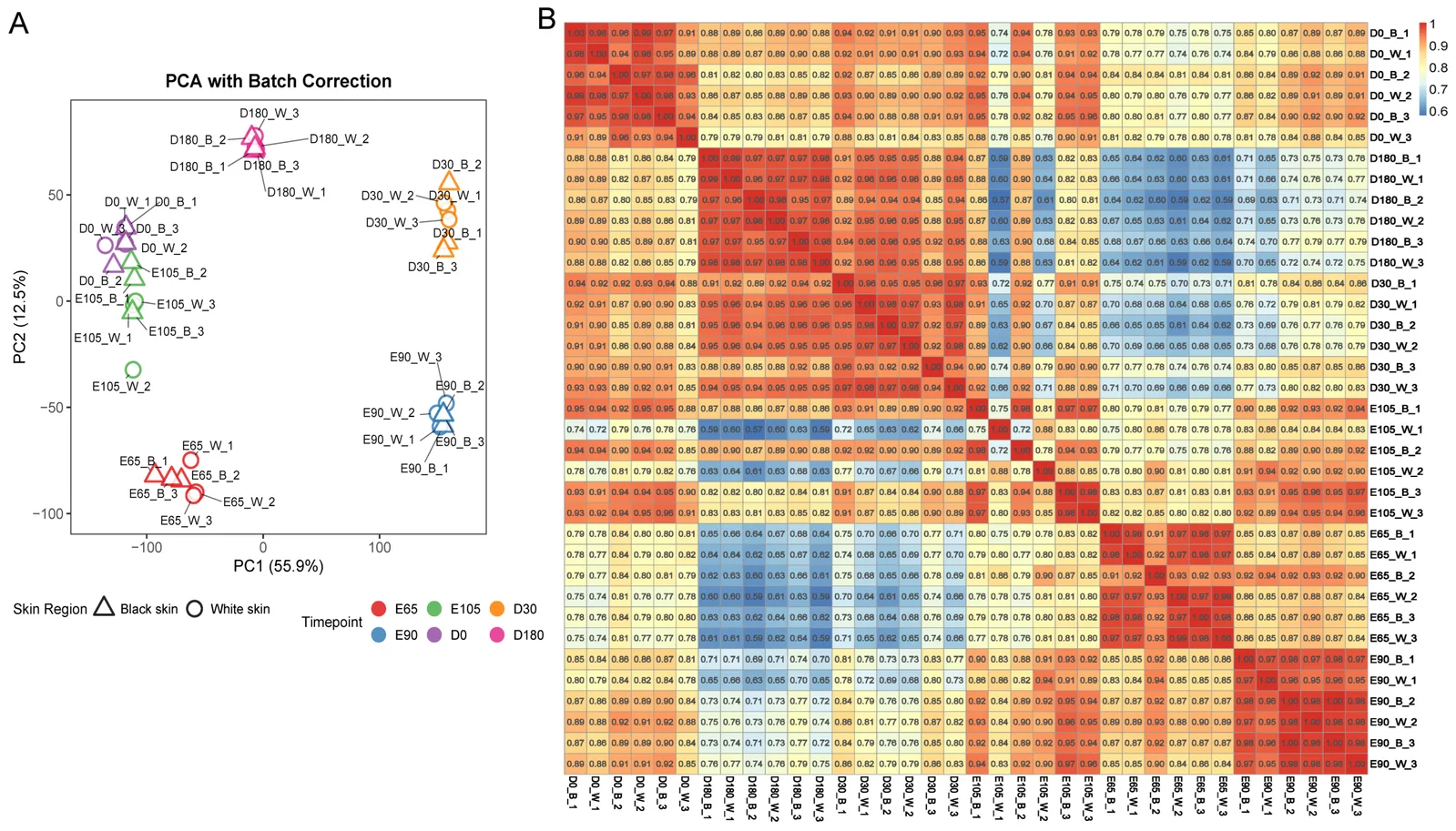
Principal component analysis and sample correlation analysis of black and white skin transcriptomes. (**A**) Principal component analysis plot of 36 black and white skin RNA-seq samples. Each point represents one biological replicate; shapes indicate skin type (triangles, black skin; circles, white skin), and colors indicate developmental stages. (**B**) Sample-to-sample Pearson correlation heatmap based on FPKM-normalized gene expression values. Biological replicates from the same developmental stage and skin type generally showed higher correlations and closer clustering.

All 36 skin samples generated high-quality strand-specific RNA-seq data. The Q30 values exceeded 92.86%, the average GC content was 51.03%, and more than 95.15% of clean reads were uniquely mapped to the *Sus scrofa* 11.1 reference genome (Supplementary Table S3), indicating that the sequencing data were suitable for downstream transcriptomic analyses. Principal component analysis (PCA) showed that skin samples were primarily separated according to developmental stage (Figure 3A). PC1 explained 55.9% of the total variance, while PC2 explained 12.5%. Samples from the same developmental stage tended to cluster together, and biological replicates within the same group showed close clustering. Sample correlation analysis showed that biological replicates generally exhibited high correlations, with Pearson correlation coefficients mostly exceeding 0.7 (Figure 3B). Hierarchical clustering based on standardized expression profiles further supported the PCA results, showing that samples were largely grouped according to developmental stage. Biological replicates from the same developmental stage and coat color were generally clustered together, suggesting acceptable within-group consistency. Together, these results indicate that the RNA-seq dataset was suitable for subsequent analyses of developmental and coat color-associated transcriptomic differences.

### 3.3. Stage-Specific Differential Gene Expression Analysis Between Black and White Skin

**Figure 4:**
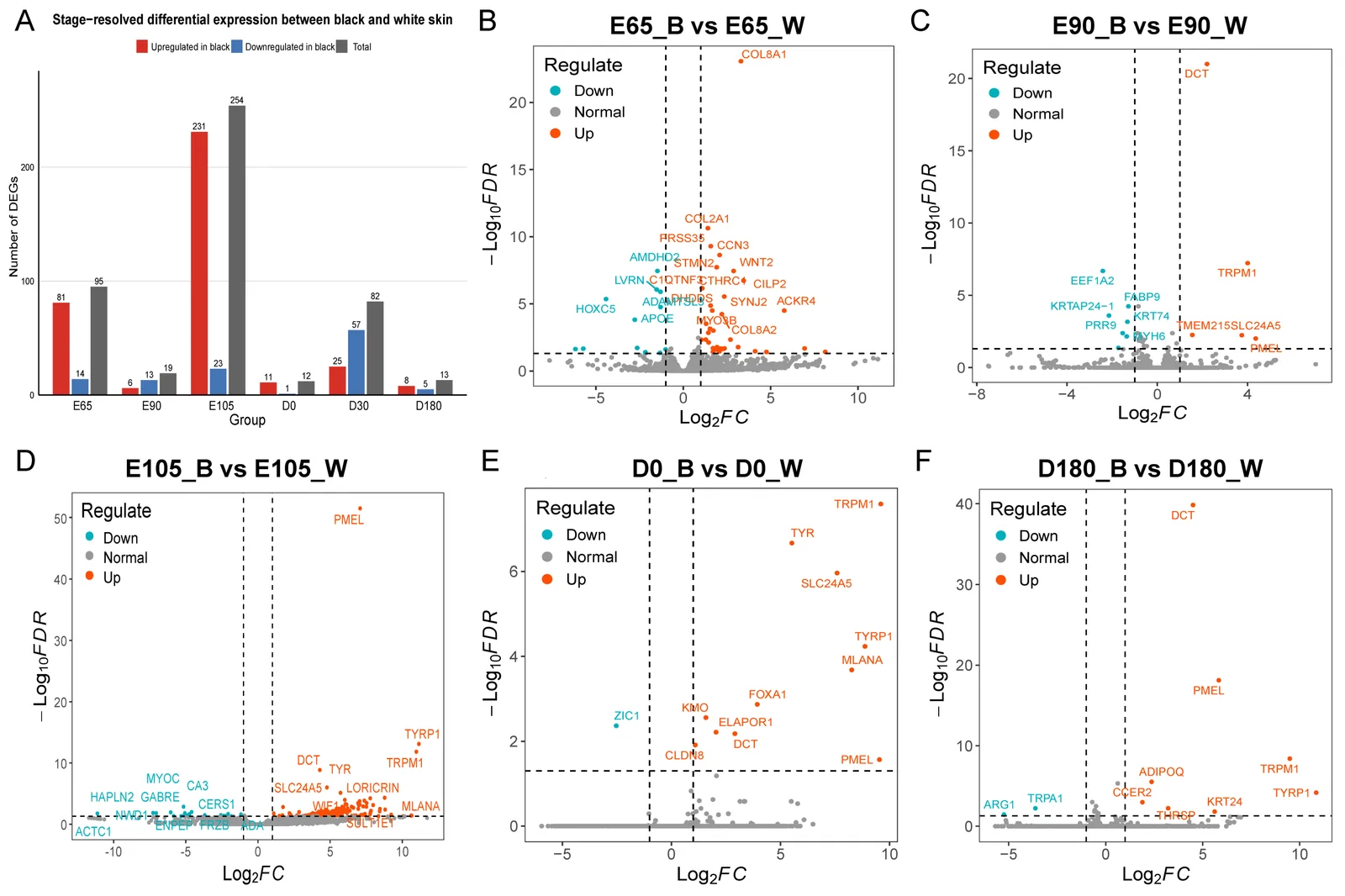
Stage-resolved paired differential expression analysis between black and white skin. (**A**) Bar plot of upregulated, downregulated, and total DEGs between black and white skin at each developmental stage, using the thresholds |log2FC| ≥ 1 and FDR < 0.05. (**B**–**F**) Volcano plots of black–white differential expression at selected developmental stages: E65 (**B**), E90 (**C**), E105 (**D**), D0 (**E**), and D180 (**F**). Red, green, and grey points indicate genes upregulated in black skin, downregulated in black skin, and non-significant genes, respectively. Selected significant genes are labeled.

Differential expression analysis was performed between paired black and white skin samples at each developmental stage using the criteria of |log2FC| ≥ 1 and FDR < 0.05. The paired analysis identified 95, 19, 254, 12, 82, and 13 DEGs at E65, E90, E105, D0, D30, and D180, respectively. The number of DEGs varied markedly across developmental stages, with the largest number detected at E105, followed by E65 and D30, indicating a stage-dependent pattern of transcriptional differences between black and white skin (Figure 4A). At E65, before obvious external coat-color differences were fully established, black and white skin already showed significant transcriptional differences. A total of 95 DEGs were identified, including 81 genes with higher expression and 14 genes with lower expression in black skin. The top DEGs between black and white skin were mainly related to extracellular matrix components, tissue remodeling, cell adhesion, and developmental regulatory processes, and included *CCN3*, *COL8A1*, *COL2A1*, *LAMA1*, and *WNT2* (Figure 4B). In addition, the pigmentation-associated genes DCT, PMEL, and TRPM1 showed significantly higher expression in black skin. These findings indicate that the early transcriptional differences between black and white skin involved both skin development and extracellular matrix-related processes, as well as emerging pigmentation-associated expression differences.

By E90, the number of black–white DEGs was low, but several pigmentation-related genes were already differentially expressed. Notably, *DCT*, *TRPM1*, *SLC24A5*, and *PMEL* showed higher expression in black skin (Figure 4C), indicating that pigmentation-associated transcriptional differences were already detectable at this stage. This pigmentation-related signature became more prominent at E105, when 254 DEGs were identified, including 231 genes with higher expression in black skin. At this stage, multiple core melanogenesis- and melanosome-associated genes, including *PMEL*, *TYRP1*, *TRPM1*, *DCT*, *TYR*, *SLC24A5*, and *MLANA*, were significantly upregulated in black skin (Figure 4D). These results indicate that E105 represents a major embryonic stage of black–white divergence in pigmentation-associated gene expression. At D0, D30, and D180, 12, 82, and 13 DEGs were identified, respectively. Several pigmentation-associated genes also showed higher expression in black skin during postnatal development (Figure 4E,F; Supplementary Figure S1C).

To characterize developmental transcriptomic dynamics within each skin type, DEGs were identified between adjacent developmental stages in black and white skin, respectively (Supplementary Figure S1A,B). In black skin, the largest number of DEGs was detected between E65 and E90, with a total of 4305 DEGs, followed by the E90-to-E105 transition with 1635 DEGs, the E105-to-D0 transition with 1291 DEGs, the D0-to-D30 transition with 1698 DEGs, and the D30-to-D180 transition with 1200 DEGs. A similar but not identical pattern was observed in white skin. In white skin, the transition from E65 to E90 also showed the largest number of DEGs, reaching 4936, whereas only 296 DEGs were detected between E90 and E105. Subsequent comparisons identified 1190 DEGs between E105 and D0, 1825 between D0 and D30, and 1084 between D30 and D180. Overall, these results indicate that both black and white skin underwent significant transcriptomic remodeling during the transition from E65 to E90, with additional stage-dependent changes occurring around birth and during postnatal development. The distinct DEG patterns between black and white skin suggest that the two skin types differed in the timing and magnitude of developmental transcriptional changes.

### 3.4. Temporal Expression Clustering and Pathway Activity Analysis Reveal Developmental Dynamics of Pigmentation-Related Programs in Black Skin

**Figure 5:**
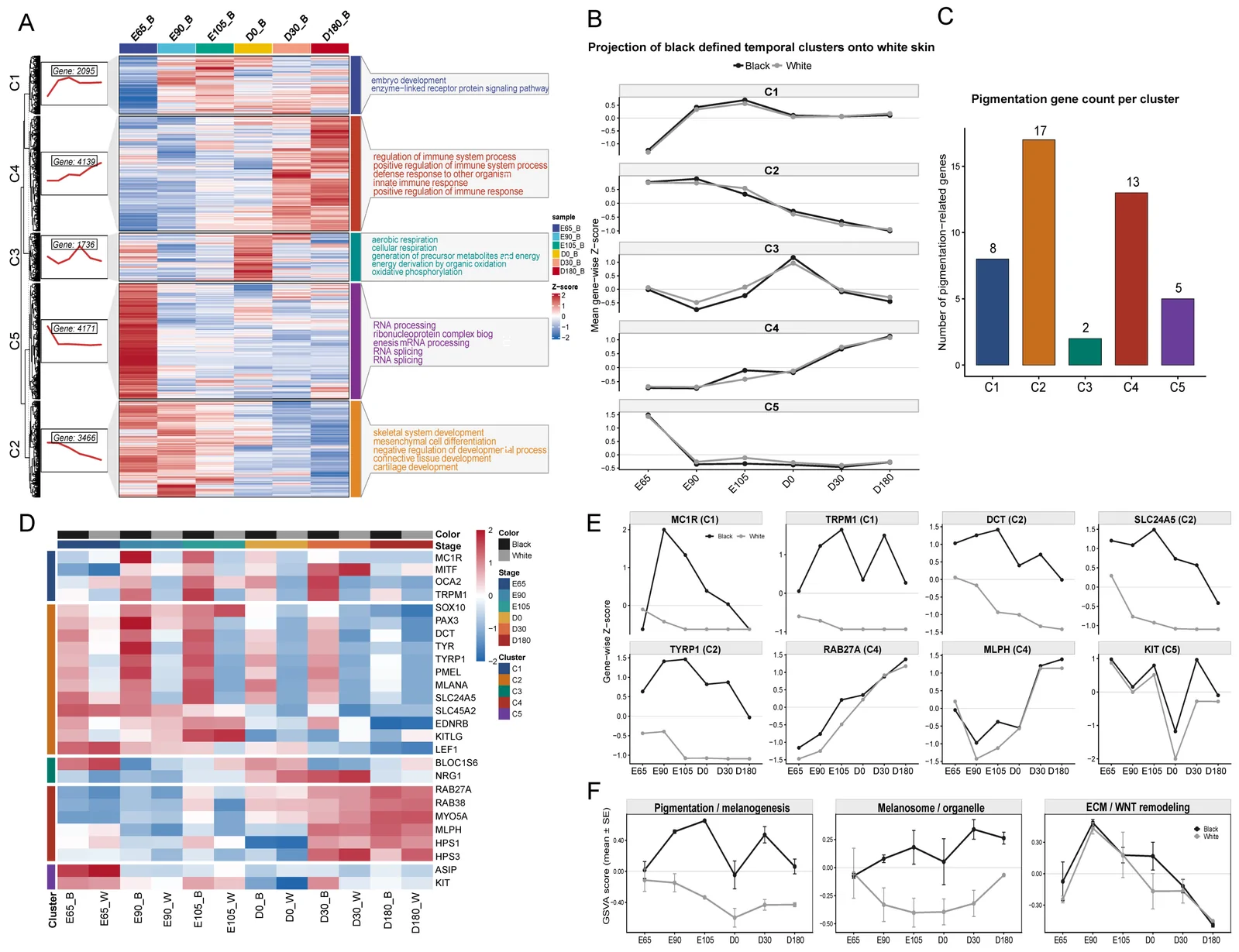
Temporal clustering and pathway activity analysis of pigmentation-associated transcriptional programs. (**A**) ClusterGVis heatmap of five temporal expression clusters identified from the average expression profiles of black skin samples across six developmental stages, with representative enriched biological processes annotated for each cluster. (**B**) Projection line plots of black skin-defined temporal clusters onto white skin samples. (**C**) Bar plot of curated pigmentation-related genes assigned to each temporal expression cluster. (**D**) Heatmap of representative pigmentation-related genes across developmental stages from E65 to D180 and coat colors. (**E**) Temporal expression line plots of key pigmentation-related genes in black and white skin. (**F**) GSVA score plots of curated gene sets related to pigmentation/melanogenesis, melanosome/organelle function, and ECM/WNT remodeling across developmental stages and skin colors, presented as the mean ± SEM (*n* = 3 biological replicates per skin color at each developmental stage).

To characterize the temporal transcriptional programs associated with black skin development, we performed ClusterGVis-based clustering using the expression profiles of black skin samples across six developmental stages. Five major temporal expression clusters were identified, designated C1–C5 (Figure 5A). These clusters represented distinct developmental expression patterns. C5 showed high expression at E65 followed by a rapid decline, whereas C1 and C2 were mainly active during embryonic stages. C3 displayed a transient increase around birth, while C4 showed increased activity during postnatal development. Functional annotation indicated that these clusters were associated with diverse biological processes, including embryonic development, RNA processing, energy metabolism, immune regulation, and tissue maturation. To determine whether these black skin-defined temporal programs were also present in white skin, the same cluster-defined gene sets were projected onto white skin samples at the corresponding developmental stages. The projected trajectories showed broadly similar temporal patterns of C1–C5 between black and white skin (Figure 5B), suggesting that the two skin types share major developmental transcriptional programs. However, differences in expression intensity were observed in specific clusters, indicating that coat color-associated transcriptional divergence may occur within a shared developmental framework rather than through complete remodeling of the global developmental trajectory.

Pigmentation-associated genes were unevenly distributed across the five clusters and were mainly concentrated in C2 and C4 (Figure 5C). C2 contained multiple canonical melanogenesis and melanocyte-lineage genes, including *TYR*, *TYRP1*, *DCT*, *PMEL*, *MLANA*, *SOX10*, *SLC24A5*, and *SLC45A2*, whereas C4 included genes related to melanosome transport and organelle trafficking, such as *RAB27A*, *RAB38*, *MYO5A*, *MLPH*, *HPS1*, and *HPS3*. Consistently, the heatmap and representative gene trajectories showed stronger expression of melanogenesis-related genes in black skin during embryonic stages, followed by sustained or increased expression of melanosome transport-related genes during postnatal development (Figure 5D,E). GSVA-based gene-set activity analysis further supported these observations. The pigmentation/melanogenesis and melanosome/organelle gene sets showed higher activity in black skin than in white skin at specific developmental stages, with elevated activation around E105 and sustained activity during postnatal development (Figure 5F). In contrast, the ECM/WNT remodeling gene set displayed a less coat color-specific pattern, consistent with its broader role in skin morphogenesis and tissue remodeling. Together, these results indicate that, within a shared developmental transcriptional framework, black skin exhibited higher expression and gene-set activity of melanogenesis- and melanosome-related programs than white skin at specific developmental stages.

### 3.5. Weighted Gene Co-Expression Network Analysis Reveals Developmental Stage-Associated Modules Linked to Pigmentation and Skin Maturation

**Figure 6:**
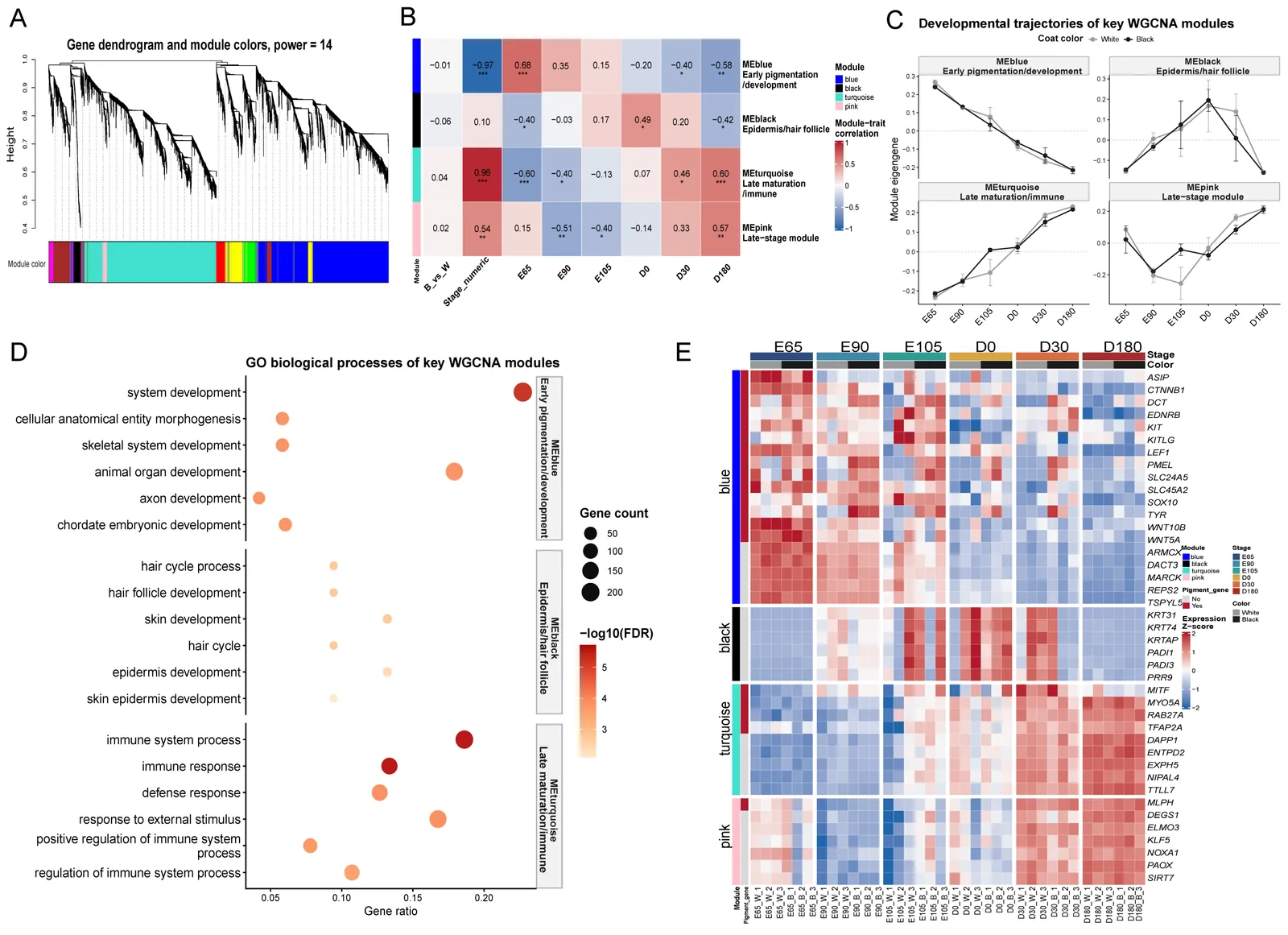
WGCNA identifies developmental stage-associated co-expression modules related to pigmentation and skin maturation. (**A**) Gene dendrogram and module color assignment from WGCNA signed network analysis using a soft-thresholding power of 14. (**B**) Module–trait correlation heatmap of key WGCNA module eigengenes and developmental stage- or coat color-related traits. Cell values indicate Pearson correlation coefficients. Corresponding *p* values were adjusted using the Benjamini–Hochberg method, and asterisks indicate FDR-adjusted significance: * FDR < 0.05, ** FDR < 0.01, and *** FDR < 0.001. (**C**) Line plots of key WGCNA module eigengenes in black and white skin across six developmental stages. Data are presented as mean ± SEM (*n* = 3 biological replicates per skin color at each developmental stage). (**D**) GO biological process enrichment bubble plot of key WGCNA modules. Dot size indicates the number of genes enriched in each GO term, and color intensity represents enrichment significance, shown as −log10(FDR). (**E**) Heatmap of hub genes from key WGCNA co-expression modules across developmental stages from E65 to D180.

To identify co-expression programs associated with transcriptional activity related to skin development and pigmentation, we performed a weighted gene co-expression network analysis using 36 skin samples. A soft-thresholding power of 14 was selected for signed network construction, and sample clustering based on WGCNA input genes showed no obvious outlier samples (Supplementary Figure S2A,B). The gene dendrogram showed that the skin transcriptome was divided into several distinct co-expression modules (Figure 6A). Module–trait correlation analysis revealed that the selected modules were primarily associated with the developmental stage, not just coat color. MEblue showed a significant negative correlation with developmental progression and was positively associated with early embryonic stages, particularly E65 and E90. However, MEturquoise showed a significant positive correlation with developmental progression and was more strongly associated with later stages, especially D30 and D180. MEPink was associated with skin developmental stages and also showed a positive correlation with D180. MEblack showed relatively high correlations around D0. Additional module eigengene trajectories are shown in Supplementary Figure S2C.

GO and KEGG enrichment analyses were performed to further characterize the biological functions of the developmental stage-associated modules (Figure 6C,D; Supplementary Figure S2D). MEblue, which was highly associated with early embryonic stages, was enriched for developmental and morphogenetic processes, suggesting that this module reflects early skin developmental programs. MEblack was enriched for skin development, epidermis development, hair follicle development, and hair cycle-related processes, consistent with its potential involvement in epidermal and follicular maturation around the perinatal stage. MEturquoise, which was associated with later developmental stages, was enriched for immune system processes, defense response, response to external stimuli, and regulation of immune responses, suggesting a potential role in postnatal skin maturation and immune-associated activity. MEpink also showed increased module eigengene expression at later stages and was associated with late-stage skin maturation-related processes.

To further illustrate the representative hub genes in the key co-expression modules, we created a heatmap (Figure 6E). Notably, the MEblue module contains two types of genes: one type includes genes related to early skin development and morphogenesis, such as *WNT10B*, *WNT5A*, *MARCKS*, and *REPS2*; the other type includes genes related to the melanocyte lineage and pigmentation, such as *DCT*, *TYR*, *EDNRB*, *SLC24A5*, *PMEL*, *SOX10*, *KIT*, and *SLC45A2*. This result suggests that pigmentation-related genes do not form an isolated “melanosis-specific module,” but are embedded in the co-expression program of early skin development. Meanwhile, genes related to melanosome transport, organelle transport, and later pigmentation processes, such as *MYO5A*, *RAB27A*, *TFAP2A*, and *MLPH*, are mainly distributed in the late-stage developmental modules. These results indicate that the expression pattern of pigmentation-related genes in the skin of Bama miniature pigs is closely related to the co-expression network associated with developmental stages and is distributed in modules related to early embryonic development, epidermal/hair follicle maturation, and postnatal skin maturation.

### 3.6. qRT-PCR Analysis of Selected Pigmentation-Associated Candidate Genes

**Figure 7:**
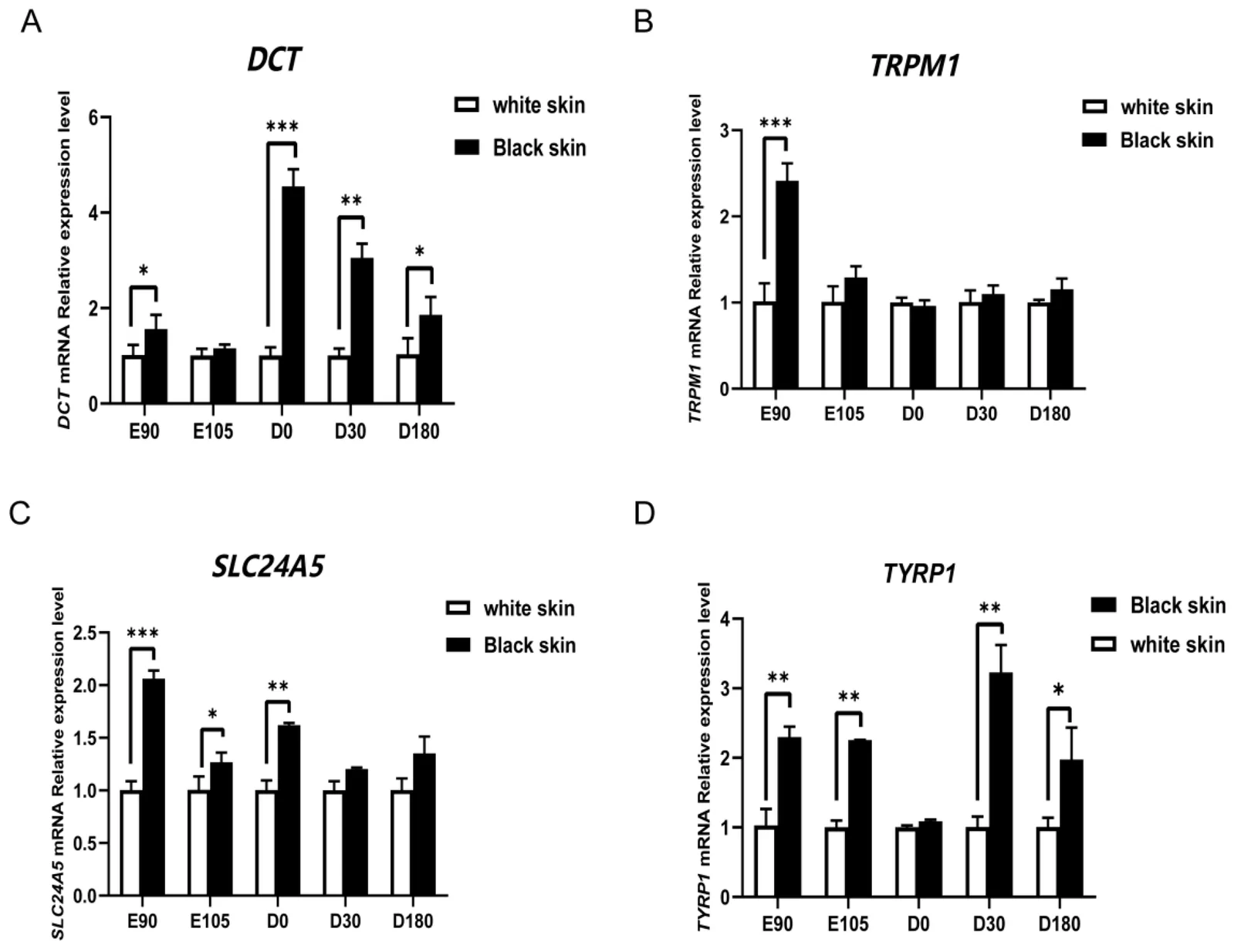
qRT-PCR analysis of selected pigmentation-associated candidate genes. (**A**–**D**) Relative mRNA expression levels of DCT (**A**), TRPM1 (**B**), SLC24A5 (**C**), and TYRP1 (**D**) in paired black and white skin samples from Bama miniature pigs at E90, E105, D0, D30, and D180. Relative expression was calculated using stage-matched white skin as the calibrator. Data are presented as mean ± SEM (*n* = 3 biological replicates per developmental stage). Statistical significance was assessed using two-way ANOVA, with skin color treated as the repeated-measures factor, followed by Bonferroni’s multiple-comparisons test for comparisons between black and white skin within each developmental stage. * *p* < 0.05, ** *p* < 0.01, and *** *p* < 0.001.

To validate the RNA-seq expression patterns of selected pigmentation-related genes, the four candidate genes *DCT*, *TRPM1*, *SLC24A5*, and *TYRP1* were examined by qRT-PCR at representative developmental stages from E90 to D180, using stage-matched white skin as the control. These genes generally showed higher relative expression levels in black skin than in white skin at one or more developmental stages, although the magnitude and timing of the differences varied among genes (Figure 7). The qRT-PCR results were broadly consistent with the RNA-seq expression patterns, supporting the consistency of the expression trends observed for these candidate genes and confirming their black skin-biased expression at specific developmental stages.

## 4. Discussion

The skin is one of the largest mammalian organs and contains melanocytes [29], which contribute to skin and hair pigmentation [30]. Loss or dysfunction of melanocytes can result in hypopigmentation or depigmentation [31]. Recent single-cell and spatial transcriptomic studies have provided valuable resources for characterizing skin cell composition, hair follicle development, cellular heterogeneity, and the local skin microenvironment [32,33]. In pigs, previous studies have characterized developmental changes in skin cell populations, hair placode-associated cell types, and molecular remodeling during skin and hair follicle development. However, most of these studies have focused on developmental skin atlases and hair follicle formation, whereas the developmental timing and transcriptional basis of regional black–white skin differences within the same breed remain insufficiently understood [34].

Guangxi Bama miniature pigs constitute an inbred population with a relatively stable genetic background and a characteristic regional black–white skin phenotype, making this population a useful model for studying skin pigmentation differences within the same breed. In this study, we performed integrated histological and strand-specific transcriptomic analyses of paired black and white skin regions from Guangxi Bama miniature pigs across six embryonic and postnatal developmental stages. All samples were collected from female pigs, and paired black and white skin samples were obtained from the same posterior head/neck region of each individual. The overall sampling design, combining within-animal paired sampling from the same anatomical region with the use of female animals only, helped reduce variation associated with genetic background, sex, inter-individual differences, and anatomical site. Overall, our analyses revealed three main findings: developmental stage was the dominant factor shaping the skin transcriptome; molecular differences between black and white skin were detectable before regional pigmentation became fully apparent; and pigmentation-associated transcriptional programs were particularly prominent during late embryonic development, especially at E105.

PCA, sample correlation analysis, and WGCNA collectively supported the dominant effect of developmental progression on the skin transcriptome. H&E staining showed that skin structure gradually matured during development, with progressive epidermal stratification, dermal organization, and hair follicle formation. At E65, the distribution of darkly stained structures differed between black and white skin regions, with these structures observed in the epidermal and perifollicular regions of black skin. However, because H&E staining is not melanin-specific, these structures could not be conclusively identified as melanin deposits or melanocyte-associated structures. At the transcriptomic level, DEGs between black and white skin at E65 were mainly enriched in extracellular matrix organization, cell adhesion, cell motility, tissue remodeling, and developmental regulation. Meanwhile, hub genes in the E65-associated MEblue module included genes related to both early skin development and pigmentation. Thus, pigmentation-related genes at E65 may be embedded within co-expression programs related to skin development and tissue remodeling rather than forming a distinct melanogenesis-specific module. Together, these results suggest that molecular divergence between black and white skin may begin before the regional pigmentation phenotype becomes fully apparent and that pigmentation-associated transcriptional differences may become increasingly prominent as embryonic skin development progresses.

From E90 to E105, epidermal stratification and hair follicle development became more apparent, and H&E sections showed differences in the distribution of darkly stained structures between black and white skin. During the same period, pigmentation-related genes, including *DCT*, *PMEL*, *TRPM1*, *TYR*, *TYRP1*, *SLC24A5*, and *MLANA*, showed higher expression in black skin than in white skin. This period may therefore represent a key developmental window during which pigmentation-associated transcriptional differences become particularly prominent at the tissue level. This developmental window overlaps with previous reports indicating that pig hair follicle development begins around E37 and continues until P45, with E85 and E107 representing important stages of follicular differentiation [35]. The increase in pigmentation-associated gene expression during E90–E105 coincided with epidermal and hair follicle development, suggesting that these processes may be temporally coordinated [36].

At the functional level, black skin pigmentation in Bama miniature pigs appears to reflect the coordinated regulation of multiple pigmentation-related processes rather than isolated changes in a single gene. *TYR*, *TYRP1*, and *DCT* participate directly in melanin synthesis, whereas *PMEL* and *MLANA* are involved in melanosome maturation and function. *SLC24A5* is associated with melanosomal ion homeostasis and melanosome maturation, while *TRPM1* is linked to melanocyte biology and cation-channel-related signaling [37,38]. In addition, *MC1R*, *KIT*, *KITL* [39], and *EDNRB* [40] are involved in melanocyte signaling, survival, differentiation, and pigmentation regulation. The qRT-PCR analysis of *DCT*, *TRPM1*, *SLC24A5*, and *TYRP1* across representative developmental stages from E90 to D180 further supported the RNA-seq-based expression patterns. WGCNA further suggested that MEblack was associated with epidermal and hair follicle development, whereas MEturquoise and MEpink were more closely related to later skin maturation and immune-associated processes. Together, these findings suggest that pigmentation-associated transcriptional differences in black skin involve coordinated changes in melanin synthesis, melanosome biology, ion transport, melanocyte regulatory signaling, and skin structural maturation [41,42].

Despite these findings, several limitations should be acknowledged. First, bulk RNA-seq cannot distinguish whether expression differences reflect changes in melanocyte abundance, altered gene expression within melanocytes, or shifts in other skin-resident cell populations. Future single-cell RNA sequencing could help distinguish these possibilities by comparing the relative abundance of melanocytes between paired black and white skin samples and identifying melanocyte-specific differential expression. Recent single-cell profiling of primary human epidermal melanocytes has revealed transcriptional heterogeneity across anatomical sites, developmental ages, and skin tones, supporting the utility of this approach for resolving melanocyte-specific states and transcriptional programs [43]. Second, H&E staining provides morphological information but is not melanin-specific; therefore, Fontana–Masson staining, melanocyte marker immunohistochemistry, or melanosome-level ultrastructural analysis would provide more specific histological validation. Third, qRT-PCR validation was performed using representative developmental stages from E90 to D180, but E65 was not included because no RNA, cDNA, or tissue material from this stage remained after completion of the RNA-seq and related analyses. Additional samples and finer developmental time points will therefore be needed to characterize earlier pigmentation-associated changes.

Fourth, the candidate genes in this study were identified on the basis of differential expression, temporal expression patterns, co-expression network associations, and pigmentation-related functional annotations. These analyses support their potential involvement in regional skin pigmentation but provide associative rather than causal evidence. CRISPR/Cas9-mediated genetic perturbation, pharmacological inhibition where applicable, and melanocyte-based functional assays will be needed to establish their causal roles and underlying mechanisms. In addition, spatial transcriptomics and chromatin accessibility profiling may further clarify the cell type-specific regulatory mechanisms underlying regional pigmentation in Bama miniature pigs. Given the high anatomical and physiological similarities between Bama miniature pig skin and human skin [44,45,46], this model may provide a valuable reference for comparative skin biology, pigmentation-related mechanisms, and biomedical studies of human skin.

## 5. Conclusions

This study performed integrated histological and strand-specific transcriptomic analyses of black and white skin regions in Guangxi Bama miniature pigs across multiple developmental stages. The results showed that developmental stage was the major factor shaping skin transcriptomic variation, whereas differences between black and white skin regions exhibited clear stage-dependent characteristics. Molecular differences between black and white skin regions were already detectable at E65 and were mainly associated with early skin development, extracellular matrix organization, tissue remodeling, and pigmentation-related co-expression programs. The combined histological observations and transcriptomic findings suggest that regional differences between black and white skin begin to emerge during embryonic development, become more pronounced during late embryogenesis, and persist after birth. Pigmentation-associated transcriptional differences became particularly prominent during late embryonic development, especially at E105. Genes related to melanogenesis, melanosome function, ion transport, and pigmentation signaling, including *DCT*, *TRPM1*, *SLC24A5*, and *TYRP1*, showed black-skin-biased expression at specific developmental stages. Overall, this study provides developmental transcriptomic resources and candidate genes for understanding regional skin pigmentation in Bama miniature pigs, laying a foundation for future studies on pigmentation mechanisms and pig breeding.

## Data Availability

Raw strand-specific RNA-seq data are available in the NCBI BioProject database (BioProject ID: PRJNA1490402).

## Supplementary Materials

The following supporting information can be downloaded at https://www.mdpi.com/article/10.3390/ani16162565/s1. Table S1: Body weight statistics of Guangxi Bama miniature pigs at different developmental stages; Table S2: All primer sequences used in this paper; Table S3: Summary of RNA-seq data quality and sequencing statistics; Figure S1: Developmental DEG dynamics in black and white skin and D30 black−white differential expression; Figure S2: WGCNA network construction, developmental dynamics of candidate module eigengenes, and KEGG enrichment analysis.
