# Supplementary material for: Histological and Strand-Specific Transcriptomic Analyses Reveal Developmental Dynamics and Pigmentation-Associated Candidate Genes in Black and White Skin Regions of Bama Miniature Pigs
Source: Animals (Basel). 2026 Aug 17;16(16):2565. doi: 10.3390/ani16162565 (PMC13508850; doi:10.3390/ani16162565)
Supplement: Supplementary file 1 [file animals-16-02565-s001.zip › animals-4454297-supplementary.pdf]

Supplementary Table S1. Body weight statistics of Guangxi Bama miniature pigs at different developmental stages

Day	Weight (kg)
E65	$0.08±0.004^{d}$
E90	$0.22±0.009^{d}$
E105	$0.44±0.014^{c}$
D0	$0.57±0.092^{c}$
D30	$3.30±0.236^{b}$
D180	$19.33±0.507^{a}$

Note: Values sharing the same superscript letter within the same column are not significantly different ($p > 0.05$), whereas values with different superscript letters are significantly different ($p < 0.05$).

Supplementary Table S2. All primer sequences used in this paper

Genes	Primer sequences (5'→3')	Annealing temperature/°C	Product length/bp	Applications
DCT	F: ACCCCACACCCTGACTA	55.98	115	qRT-PCR
	R: GGAGCCACACAAATAAAT	50.06		
TRPM1	F: CACCCACTCTGTCCCATCAC	59.76	165	qRT-PCR
	R: TGCGTGTCCACCTGCTTATT	60.11		
SLC24A5	F: GTCACCCAAGGGCTACAGGA	61.20	143	qRT-PCR
	R: GGCCATGAGCATGTAGAGGAT	59.65		
TYRP1	F: CCCTCAGTTCGTCATTG	51.96	182	qRT-PCR
	R: CTTCGTGAGAGAAATCCA	51.20		
ACTB	F: CCACGAAACTACCTTCAACTCC	58.94	132	qRT-PCR
(β-actin)	R: GTGATCTCCTTCTGCATCCTGT	59.83		

Supplementary Table S3. Summary of RNA-seq data quality and sequencing statistics

Sample	Raw Reads	Clean Reads	Raw Base(G)	Clean Base(G)	Effective(%)	Q20(%)	Q30(%)	GC(%)
E65_B_1	57,021,420	55,144,300	8.55	8.27	96.71	96.73	92.31	45.87
E65_B_2	49,182,976	48,615,594	7.38	7.29	95.80	99.03	97.17	48.81
E65_B_3	54,513,934	52,331,904	8.18	7.85	95.77	97.93	94.43	43.99
E90_B_1	41,499,686	39,108,428	6.22	5.87	94.24	97.51	93.76	51.66
E90_B_2	40,882,662	39,005,196	6.13	5.85	95.41	97.43	93.54	51.96
E90_B_3	45,769,220	43,145,038	6.87	6.47	94.27	97.36	93.36	51.01
E105_B_1	49,884,060	47,291,658	7.48	7.09	94.80	97.46	93.55	51.32
E105_B_2	43,629,704	41,631,868	6.54	6.24	95.42	97.54	93.76	50.99
E105_B_3	67,736,312	64,973,420	10.16	9.75	95.92	97.48	93.52	50.68
D0_B_1	44,235,726	42,043,438	6.64	6.31	95.04	97.59	93.81	51.21
D0_B_2	43,746,430	41,569,386	6.56	6.24	95.02	97.59	93.93	50.39
D0_B_3	42,486,280	40,288,410	6.37	6.04	94.83	97.42	93.46	50.90
D30_B_1	45,701,668	43,647,984	6.86	6.55	95.51	97.44	93.56	51.71
D30_B_2	46,211,288	43,890,314	6.93	6.58	94.98	97.43	93.57	51.07
D30_B_3	44,590,296	42,281,366	6.69	6.34	94.82	97.49	93.70	50.92
D180_B_1	46,387,302	44,049,226	6.96	6.61	94.96	97.52	93.64	50.40
D180_B_2	43,911,098	41,752,232	6.59	6.26	95.08	97.56	93.78	50.09
D180_B_3	41,748,974	39,861,916	6.26	5.98	95.48	97.65	93.98	50.53
E65_W_1	76,180,868	74,157,948	10.43	10.12	97.34	98.13	94.75	48.06
E65_W_2	57,496,316	55,282,132	8.62	8.29	96.15	97.99	94.51	47.58
E65_W_3	47,712,820	45,988,802	7.16	6.90	96.39	97.88	94.21	47.33
E90_W_1	40,943,488	38,737,782	6.14	5.81	94.61	97.61	94.01	51.75
E90_W_2	53,891,020	51,515,020	8.08	7.73	95.59	97.40	93.28	51.16
E90_W_3	40,108,458	38,547,038	6.02	5.78	96.11	97.35	93.33	51.2
E105_W_1	46,115,676	44,353,034	6.92	6.65	96.18	97.62	93.86	51.54
E105_W_2	40,321,700	38,757,286	6.05	5.81	96.12	97.43	93.54	51.28
E105_W_3	46,820,840	44,604,232	7.02	6.69	95.27	97.56	93.79	51.14
D0_W_1	45,858,526	43,877,350	6.88	6.58	95.68	97.60	93.94	51.75
D0_W_2	45,533,240	43,386,082	6.83	6.51	95.28	97.55	93.78	51.48
D0_W_3	43,146,722	40,501,452	6.47	6.08	93.87	97.48	93.66	50.67
D30_W_1	40,154,178	37,707,640	6.02	5.66	93.91	97.53	93.67	50.28
D30_W_2	42,882,630	40,891,826	6.43	6.13	95.36	97.14	92.86	51.22
D30_W_3	40,181,174	38,421,916	6.03	5.76	95.62	97.47	93.64	51.2
D180_W_1	39,820,374	37,845,074	5.97	5.68	95.04	97.46	93.55	50.73
D180_W_2	43,645,794	41,696,172	6.55	6.25	95.53	97.57	93.79	50.13
D180_W_3	43,963,518	41,593,000	6.59	6.24	94.61	97.49	93.66	50.48

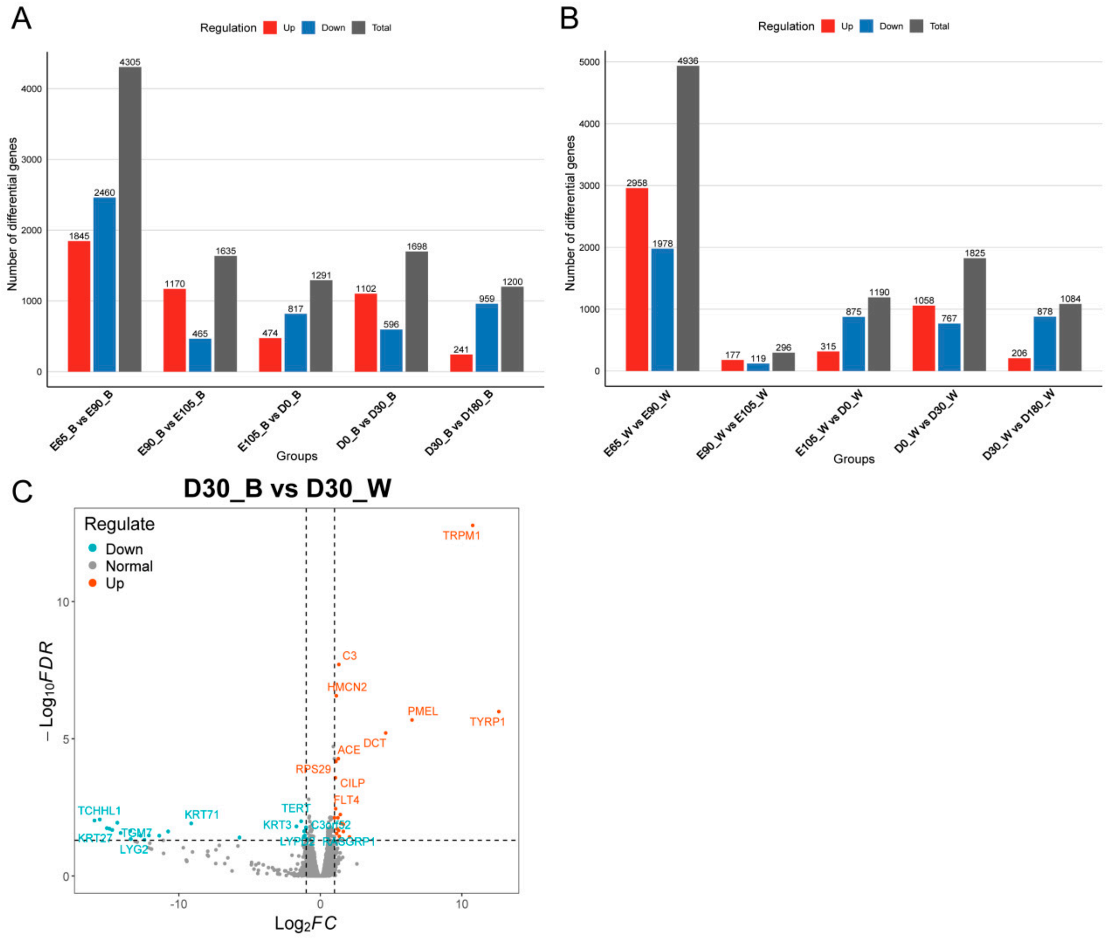

Supplementary Figure S1. Developmental DEG dynamics in black and white skin and D30 black-white differential expression. (A) Bar plot of the number of differentially expressed genes (DEGs) between adjacent developmental stages in black skin, including upregulated, downregulated, and total DEGs identified using $|\log_2 FC| \geq 1$ and FDR < 0.05. (B) Upregulated, downregulated, and total DEGs between adjacent developmental stages in white skin, identified using the same thresholds. (C) Volcano plot of paired black versus white skin differential expression at D30, with animal identity included as a blocking factor. Red points indicate genes upregulated in black skin, green points indicate genes downregulated in black skin, and grey points indicate non-significant genes.

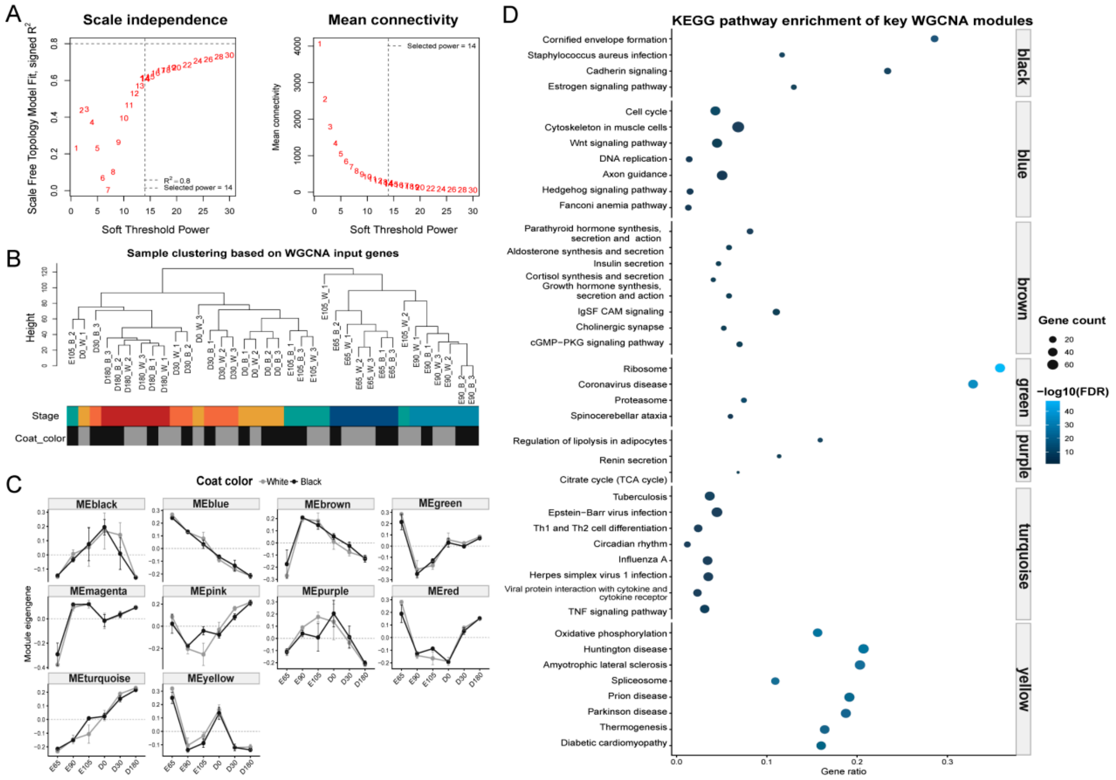

Supplementary Figure S2. WGCNA network construction, developmental dynamics of candidate module eigengenes, and KEGG enrichment analysis. (A) Soft-thresholding power analysis showing the scale-free topology fit index and mean connectivity across different power values. The horizontal dashed line indicates the reference scale-free topology fit threshold of 0.80, and the vertical dashed line indicates the selected soft-thresholding power of 14. A soft-thresholding power of 14 was selected as a balance between improving the approximation to scale-free topology and retaining sufficient network connectivity. (B) WGCNA sample clustering dendrogram with developmental stage and coat color. (C) Line plots of module eigengenes for selected candidate WGCNA modules in black and white skin across six developmental stages. Data are presented as mean ± SE. (D) KEGG enrichment profiles of selected WGCNA modules. Module colors are shown as facet labels on the right. Gene ratio is shown on the x-axis; dot size indicates gene count, and dot color represents -$\log_{10}$(FDR).
